# Circulating Relaxin-1 Level Is a Surrogate Marker of Myocardial Fibrosis in HFrEF

**DOI:** 10.3389/fphys.2019.00690

**Published:** 2019-06-04

**Authors:** Judit Simon, Endre Nemeth, Annamaria Nemes, Maria Husveth-Toth, Tamas Radovits, Gabor Foldes, Loretta Kiss, Zsolt Bagyura, Judit Skopal, Bela Merkely, Edit Gara

**Affiliations:** ^1^Heart and Vascular Center, Semmelweis University, Budapest, Hungary; ^2^Department of Anaesthesiology and Intensive Therapy, Semmelweis University, Budapest, Hungary

**Keywords:** relaxin-1, fibrosis, biomarker, HFrEF, heart transplantation, hemodynamics

## Abstract

**Introduction:** Relaxin-1 (RLN1) has emerged as a possible therapeutic target in myocardial fibrosis due to its anti-fibrotic effects. Previous randomized clinical trials investigated therapeutic role of exogenous relaxin in patients with acute-on-chronic heart failure (HF) and failed to meet clinical endpoints. Here, we aimed to assess endogenous, circulating RLN1 levels in patients with heart failure with reduced ejection fraction (HFrEF) of ischemic origin. Furthermore, we analyzed relation of RLN1 and left ventricular diastolic function, left and right ventricular fibrosis, and invasive hemodynamic measurements. Unique feature of our study is the availability of *ex vivo* human myocardial tissue.

**Methods:** Human myocardial samples were available from the Transplantation Biobank of the Heart and Vascular Center at Semmelweis University after local ethical approval and informed consent of all participants (*n* = 47). Tissue was collected immediately after heart explantations; peripheral blood was collected before induction of anesthesia. Myocardial sections were stained for Masson’s trichrome and Picrosirius red staining to quantify fibrosis. Medical records were analyzed (ECG, anthropometry, blood tests, medication, echocardiography, and invasive hemodynamic measurements).

**Results:** Average RLN1 levels in HFrEF population were significantly higher than measured in age and gender matched healthy control human subjects (702 ± 283 pg/ml in HFrEF vs. 44 ± 27 pg/ml in control *n* = 47). We found a moderate inverse correlation between RLN1 levels and degree of myocardial fibrosis in both ventricles (*r* = −0.357, *p* = 0.014 in the right ventricle vs. *r* = −0.321, *p* = 0.028 in the left ventricle with Masson’s trichrome staining). Parallel, a moderate positive correlation was found in left ventricular diastolic function (echocardiography, E/A wave values) and RLN1 levels (*r* = 0.456, *p* = 0.003); a negative correlation with RLN1 levels and left ventricular end-systolic diameter (*r* = −0.373, *p* = 0.023), and diastolic pulmonary artery pressure (*r* = −0.894, *p* < 0.001). RLN1 levels showed moderate correlation with RLN2 levels (*r* = 0.453, *p* = 0.0003).

**Conclusion:** Increased RLN1 levels were accompanied by lower myocardial fibrosis rate, which is a novel finding in our patient population with coronary artery disease and HFrEF. RLN1 can have a biomarker role in ventricular fibrosis; furthermore, it may influence hemodynamic and vasomotor activity via neurohormonal mechanisms of action. Given these valuable findings, RLN1 may be targeted in anti-fibrotic therapeutics and in perioperative care of heart transplantation.

## Introduction

Relaxin (RLN) is a polypeptide hormone that was first examined for its potential role in reproduction and pregnancy. RLN1 and 2 were reported to increase the collagen solubility in the cervix of the uterine, as well as to soften the pubic symphysis in the pregnant women (Hansell et al., 1991; Sherwood, 2004; Schwabe and Bullesbach, 2007). The RLN peptide family consists of seven members and produced by interstitial cells of various organs (Dschietzig and Stangl, 2003; Park et al., 2005; Kong et al., 2010). Further studies reported that cardiac cells are able to produce and secrete RLN and their receptors were identified in the heart (Taylor and Clark, 1994; Hsu et al., 2002). This fact has been underpinned by several studies in which RLN1-knock out mice were studied (Du et al., 2003; Samuel et al., 2004b). In these animals, large-scale fibrosis was reported in the lungs, kidneys, and left ventricle of the heart (Dschietzig et al., 2006). This fibrosis was reversible by administrating exogenous RLN (Huang et al., 2011).

Ischemic heart disease is one of the most important causes of heart failure (HF). A key mechanism of HF is cardiac remodeling caused by myocardial fibrosis and cardiomyocyte injury (Heusch et al., 2014). There are two types of myocardial fibrosis: interstitial fibrosis and replacement myocardial fibrosis (Ambale-Venkatesh and Lima, 2015). Myocardial interstitial fibrosis is defined by diffuse, disproportionate accumulation of collagen (Gonzalez et al., 2018). Replacement myocardial fibrosis is the result of post-myocardial infarction necrosis and scar formation. Cardiomyocyte injury activates fibroblasts resulting in collagen turnover and extracellular fiber deposition. Even if these mechanisms are essential in the stabilization and relative function of the injured area, they can cause abnormal stiffening and impaired ventricular function, HF, arrhythmia or even sudden cardiac death (Weber et al., 1993; Carillon et al., 2016). Clinical trials have shown that higher endogenous RLN1 levels are present in patients with HF, but these elevated levels proved to be not enough to compensate the above mentioned pathologic mechanisms (Gu et al., 2012). RLN produced by cardiac myocytes and interstitial cells has been shown to stimulate myocardial cell growth in neonatal mice. Other studies have shown that deficiency of RLN1 gene leads to cardiac fibrosis with ageing (Samuel et al., 2005) and that RLN1 and 2 reduce Type I and III collagen expression in cardiac fibroblasts (Samuel et al., 2004a; Carillon et al., 2016).

Unique feature of our study is the availability of human myocardial tissue samples (explanted failing hearts) for molecular biology and histology investigations, parallel with a high volume clinical database and clinical datasets in the perioperative period of heart transplantation surgeries. We aimed to evaluate perioperative blood tests, echocardiography, and invasive hemodynamic measurements in comparison with circulating RLN1 levels. We hypothesized that circulating RLN1 levels affect the degree of left and right ventricular myocardial fibrosis and act via the Notch signaling pathway in the failing human myocardium. To underpin our hypothesis, we quantified left and right ventricular myocardial fibrosis and its correlation with circulating RLN1 and investigated gene expression levels of RLN1 and Notch-1 signaling. Furthermore, we aimed to compare relaxin levels with echocardiography results and hemodynamic parameters in the context of postoperative vasoplegia syndrome as well.

## Materials and Methods

### Patient Population and Clinical Data

The study population comprised of 47 patients with HF with reduced ejection fraction (HFrEF), ischemic etiology and clinical criteria for heart transplantation. All of our patients were active on heart transplantation waiting list upon recruitment. The Heart and Vascular Center at Semmelweis University runs a detailed database for the Transplantation Biobank, in which full medical history, anthropometry, clinical data, medications, blood tests, echocardiography results, pharmacologic, and device therapy such as pacemaker (PM), implantable cardioverter defibrillator (ICD), cardiac resynchronization therapy (CRT), and mechanical circulatory support (MCS) data are available upon retrospective collection. For this study, end-stage HF patients with ischemic etiology were recruited. For control subjects, healthy volunteers of a cross-sectional voluntary cardiovascular screening program (Budakalász Health Examination Survey) (Kiss et al., 2018) were age and sex matched.

Blood sampling for all HFrEF patients was performed immediately before induction of the anesthesia for heart transplantation surgery. Blood sampling among healthy controls was performed at random time points on their visit of our epidemiology study. Echocardiography was performed by various number of investigators from the Heart and Vascular Center and other cardiology centers. Echocardiography data were obtained from the database of the Transplantation Biobank. Given the wide range of referring institutes for a heart transplantation, incomplete echocardiography reports existed, which limits interpretation of advanced imaging (such as TDI or speckle tracking). Echocardiography was performed at the time of candidacy and referral for the heart transplantation list. Invasive hemodynamic measurements were carried out via pulmonary artery catheterization (PAC) to assess eligibility for heart transplantation within 1 month of referral, and at multiple time points in the perioperative period. All patients had PAC and invasive hemodynamic measurements at 24 h follow-up, post-operatively.

### Biobanking Procedure

In the Transplantation Biobank of the Heart and Vascular Center at Semmelweis University, we gather various samples of the patients with end-stage heart disease who undergo heart transplantation at the Heart and Vascular Center at Semmelweis University. These samples include the explanted heart for histology and molecular biology probes, and blood samples.

The main steps of the biobanking procedure are the following: first, whole blood is drawn from recipients. After clotting and centrifugation, serum is aliquoted and stored immediately at −80°C for further evaluation. Immediately after excision of the recipients’ heart, sections of left, and right ventricles are freshly frozen in liquid nitrogen in the operation theaters; other samples from the same myocardial areas are preserved in formaldehyde. Later, freshly frozen samples are stored in −80°C and sections in formaldehyde are processed for embedding and tissue histology. The biobanking procedure is strictly organized in 24/7 and follows standard operation protocols.

### ELISA Measurements and Biomarkers

Circulating RLN1 levels were measured from collected serum with solid phase sandwich ELISA (R&D Systems, Minneapolis, MN, United States, DY3257). Experimental steps strictly followed product catalog guide. Relaxin-2 (RLN2) levels were also measured from serum (R&D Systems, DRL200). To further assess levels of standard and novel cardiac biomarkers, NT-pro-BNP, CA125 and cholinesterase were measured in our blood laboratory, on site.

### Quantitative Analysis of Cardiac Fibrosis

We evaluated the extent of myocardial fibrosis on paraffin-embedded sections of the anterior wall segments of both left and right ventricles (free wall of the right ventricle) with Masson’s trichrome (Sigma Aldrich, St. Louis, MO, United States, HT15) and Picrosirius red (*Sigma Aldrich, 365548*) staining. Both methods are gold standard in the examination of tissue fibrosis, however, they are not specific for interstitial myocardial fibrosis.

Ventricular tissue samples were collected in 4% buffered paraformaldehyde solution right after heart explant. Tissue was fixed for 24 h, room temperature. Then, tissue samples were placed in 70% ethanol. Later on, ventricular samples were dehydrated in ethanol baths, embedded in paraffin, and mounted on salinized plates. Five micrometer thick sections were obtained and stained for 1 h in Picrosirius solution (saturated picric acid, pH 2) and for 5 min in Masson’s trichrome solution, followed by differentiation with 5% phosphortungstic acid for 10 min and Aniline blue for 5 min (Sigma Aldrich, P4006 and B8563, respectively). For the washing, 0.2% acetic acid (Sigma Aldrich, 537020) was used. The stained sections were scanned and analyzed at 20× magnification (images taken at full resolution with a single image dimension set at 1,024 × 768 pixels, pixel size is 0.23 μm × 0.23 μm). The digital pictures were taken by Zeiss Axio microscope. In order to assess average myocardial fibrosis, two sections were processed from each sample and pictures of five different areas were scanned from each section (strictly capturing the right and left upper and lower extremities and the center of each section), thus altogether 20 independent images per sample were taken. The quantitative analyzes of the images were carried out with software ImageJ (bundled with 64-bit Java 1.8.0_112). For all images to be analyzed, the software applies a color transformation from the RGB to the CIE LAB color space. Then for each component (e.g., collagen fibers, cardiomyocytes) specific thresholds were applied, which identified the compartments of interest the best. After the assessment of all the components in pixel, the software presents the results in a table, including the percentage of the fibrotic area: fibrotic area/(fibrotic area + area occupied by cardiomyocytes and cardiac vasculature).

### Real-Time PCR

For quantitative real time PCR, fresh frozen interventricular septum samples of patients with end stage HF were analyzed. Total RNA was isolated with the RNeasy Mini kit (Qiagen, Venlo, Netherlands, 74104) following homogenization of all tissue samples in a Precellys^®^ Evolution homogenizer equipped with a Cryolys^®^ dry ice cooling system (Bertin Technologies, Montigny-le-Bretonneux, France). Experimental procedure strictly followed product catalog guides. RNA concentrations were measured by spectrophotometry in NanoDrop (Thermo Fisher Scientific, Waltham, MA, United States, ND2000). cDNA was reverse transcribed from extracted RNA using the High Capacity cDNA Reverse Transcription Kit (Thermo Fisher Scientific, 4368814). TaqMan Gene Expression Assays were used to quantify the mRNA expression levels of genes of interests: Relaxin-1 (Hs04194320_s1), Notch-1 (Hs01062014_m1), and ACTA-2 (Hs00426835_g1). Human glyceraldehyde 3-phosphate dehydrogenase GAPDH (Hs02758991_g1) was used as endogenous housekeeping gene. The PCR was performed with real-time PCR instrument (Applied Biosystems, Foster City, CA, United States, StepOnePlus) and the relative expressions were determined by ΔΔCt method.

### Ethics

This study was carried out in accordance with the recommendation of the Hungarian Medical Research Council [ETT TUKEB 7891/2012/EKU (119/PI/12.) and TUKEB 73/2005] and their ethical approval of standard operation protocols of the Transplantation Biobank of the Heart and Vascular Center at Semmelweis University. The healthy subjects were assessed via the Budakalasz Health Examination Survey (ETT TUKEB 8424-0/2011-EKU). All subjects gave written informed consent in keeping with the Declaration of Helsinki.

### Statistical Analyses

Samples from 47 independent patients and 36 age and sex matched healthy volunteers were used. All ELISA and PCR experiments were performed with at least three technical replicates. For statistical analyzes Student’s non-parametric *t*-test and Pearson’s correlation test were used. Data are presented as mean ± SD; *p*-values < 0.05 were considered to be statistically significant for all analyzes. Statistics and diagrams were established in GraphPad Prism 7.0.

## Results

### Demographic Data and Medical History

The mean age of the enrolled patients was 58 ± 6 years. Only 10.6% of the patients were female referring to greater prevalence of ischemic cardiomyopathy in men. All patients were on maximal tolerable doses of evidence based medications of chronic HF. Demographics, medical history are reported in Table 1. Medications are reported in Table 2.

**TABLE 1:** Demographic, laboratory, echocardiographic, and hemodynamic parameters of the enrolled patient population with HFrEF.

**Demographics and medical history**			
Age (years)	58.0±6	BSA (m^2^)	3.95±0.6
Female [*n*] (%)	5 (10.6)	ICD [*n*] (%)	36 (76.5)
Hypertension [*n*] (%)	23 (48.9)	CRT/PM [*n*] (%)	32 (68.0)
Diabetes mellitus [*n*] (%)	18 (38.2)	LVAD [*n*] (%)	2 (4.2)
BMI (kg/m^2^)	26.8±3.7	IABP [*n*] (%)	3 (6.3)
Atrial fibrillation [*n*] (%)	25 (53.1)	Hyperuricemia [*n*] (%)	16 (34.0)
COPD [*n*] (%)	3 (6.3)	GORD [*n*](%)	15 (31.9)
**Laboratory parameters**			
Creatine Clearance (ml/min/1.73 m^2^)	119.2±39.2	GGT (U/L)	139.1±131.7
Urea (mg/dl)	23.3±83.6	ALP (U/L)	159.9±121.9
eGFR (ml/min/1.73 m^2^)	67.2±67.2	Total bilirubin (μmol/L)	22.7±18.5
Glucose (mmol/L)	6.2±1.5	Leucocyte count (G/l)	8.5±2.8
Sodium (mEq/L)	135.9±3.6	Hemoglobin (g/dL)	12.8±2.1
Potassium (mEq/L)	4.4±0.5	Haematocrit (%)	38.3±5.9
SGOT (U/l)	36.7±53.6	Platelet count (G/l)	211.8±74.8
SGPT (U/l)	38.8±62.8	CRP	9.5±15.5
**Echocardiographic parameters**			
LVEF (%)	23.4±5.5	LVESV (mL)	133.3±109.0
E (cm/s)	87.0±24.5	LVEDV (mL)	166.6±154.0
E/A	2.47±1.9	RVEDD (mm)	43.2±12.5
TAPSE (mm)	16.5±4.8	horizontal RAD (mm)	53.4±20.0
E DCT (ms)	140.1±28.0	vertical RAD (mm)	49.7±10.0
LVEDD (mm)	68.6±10.5	septum DD (mm)	9.5±2.5
LVESD (mm)	60.4±11.5		
**Hemodynamic parameters**			
PVR (Dynes/sec/cm^–5^/m^2^)	215.37±158.5	diastolic PAP (mmHg)	20.65±6.5
PVR (Wood units = mmHg⋅min/L)	2.69±1.8	mean PAP (mmHg)	29.98±8.5
PCWP (mmHg)	21.76±7.15	CO (L/min)	4.02±1.0
systolic PAP (mmHg)	48.27±14.5	CI (L/min/m^2^)	1.05±0.35

*Continuous variables are reported as mean ± SD, whereas categorical variables as frequencies. Echocardiography and invasive hemodynamic measurements were obtained upon referral for heart transplantation list. Blood samples were drawn after the induction of anesthesia for heart transplantation surgery. Total patient number *n* = 47 (CI, Cardiac index; CO, Cardiac output; COPD, chronic obstructive pulmonary disease; DCT, deceleration time; DD, diastolic diameter; E, E wave; E/A, E wave/A wave; GORD, gastro-oesophageal reflux disease; IABP, intra-aortic balloon pump; LVEDD, left ventricular end-diastolic diameter; LVEDV, left ventricular end-diastolic volume; LVEF, Left ventricular ejection fraction; LVESD, left ventricular end-systolic diameter; LVESV, left ventricular end-systolic volume; PAP, Pulmonary artery pressure; PCWP, Pulmonary capillary wedge pressure; PVR, Pulmonary vascular resistance; RAD, right atrial diameter; RVEDD, right ventricular end-diastolic diameter; TAPSE, tricuspid annular plane systolic excursion).*

**TABLE 2:** Medications taken by enrolled patients with HFrEF and end-stage heart failure on heart transplantation waiting list.

**Medications**	**[n] (%)**		**[n] (%)**
**ACE inhibitors**			
Ramipril [*n*] (%)	27⁢(57.4)	Perindopril [*n*] (%)	4⁢(8.5)
Enalapril [*n*] (%)	12⁢(25.5)		
**β receptor inhibitors**			
Bisoprolol [*n*] (%)	16⁢(34.0)	Carvedilol [*n*] (%)	3⁢(6.3)
Nebivolol [*n*] (%)	12⁢(25.5)	Metoprolol [*n*] (%)	3⁢(6.3)
**Mineralocorticoid inhibitors**			
Spironolactone	29⁢(61.7)	Eplerenone	17⁢(36.1)
**Others**			
Statins	42⁢(89.3)	Platelet inhibitors	32⁢(68.0)
Amiodarone	16⁢(34.0)	Oral anticoagulation	29⁢(61.7)
Digoxin	1⁢(2.1)	Normodipine	2⁢(4.2)
Pantoprazole	38⁢(80.5)	Hydralazine	5⁢(10.6)
Nitrate	27⁢(57.4)	Anti-diabetics	12⁢(25.5)
Ivabradine	3⁢(6.3)	Trimetazidine	5⁢(10.6)
Diuretics	41⁢(87.2)		

*Of note, most of the patients required down-grading of doses and medications due to intolerance in end-stage heart failure. ACE, angiotensin converting enzyme.*

### Blood Samples and Biomarkers

**FIGURE 1:**
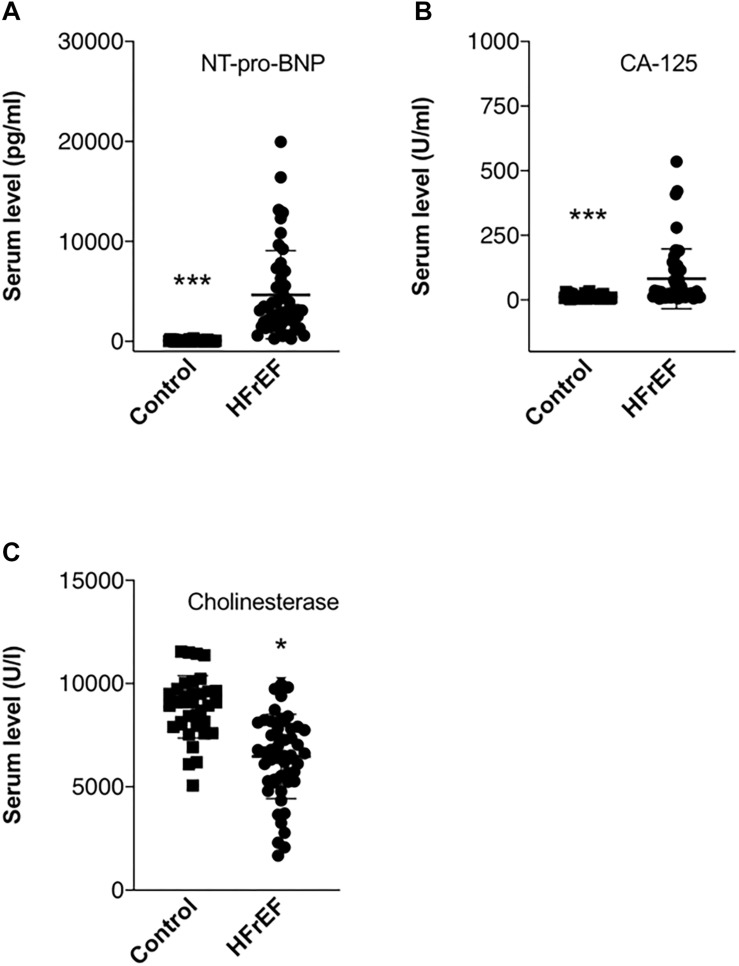
Biomarkers in end stage HFrEF and ischemic etiology. Whisker dot plots show levels of **(A)** NT-pro-BNP, **(B)** CA-125, and **(C)** cholinesterase measured from serum drawn immediately before heart transplantation surgery. Control healthy subjects were age and sexed matched, bloods were drawn at individual time points. Student’s *t* test, *n* = 47. ^*^p < 0.05, ^∗∗∗^*p* < 0.001.

**FIGURE 2:**
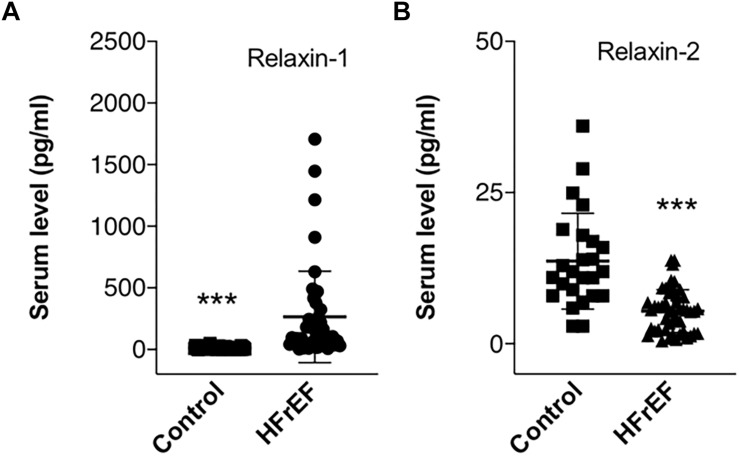
Circulating Relaxin-1 and Relaxin-2 in end stage HFrEF and ischemic etiology. Whisker dot plots show levels of **(A)** Relaxin-1 and (**B**) Relaxin-2 measured from serum drawn immediately before heart transplantation surgery. Control healthy subjects were age and sexed matched, bloods were drawn at individual time points. Student’s *t* test, *n* = 47. ^∗∗∗^*p* < 0.001.

Detailed laboratory test results can be seen in Table 1. Beside a general cardiac biomarker (NT-pro-BNP) we have also measured novel cardiac biomarkers (CA-125 and RLN); furthermore, cholinesterase levels from serum collected right before heart transplantation surgery were evaluated. Figure 1 shows cardiac biomarkers and cholinesterase levels in the HFrEF population compared with the healthy control subjects. NT-pro-BNP and also CA-125 was significantly increased in the HFrEF population. RLN1 and RLN2 levels are shown in Figure 2. Of note, we found a significantly increased level of RLN1 in patients with HFrEF; and surprisingly, RLN2 was higher in the control group. Survival at 1 year post-operatively was studied in the HFrEF population. Survival was independent of all blood sample results and biomarkers. Table 3 shows that CA-125 levels were in correlation with NT-pro-BNP levels, suggesting its cardiac biomarker feature. Furthermore, RLN1 levels correlated with RLN2 levels. Comparing pre- and postoperative (24, 48, and 72 h) blood samples, RLN and cholinesterase levels were in correlation with acute phase proteins and invasive hemodynamic parameters, such as CRP, cardiac index, and pulmonary vascular resistance (Table 3).

**TABLE 3:** Association of circulating RLN1 and RLN2, hemodynamic parameters, and acute phase proteins before and after heart transplantation surgery with ischemic etiology and HFrEF.

**Parameter**	**Mean**	**Correlation coefficient**	***p*-value**
CA-125 (pre-op)	82.9±16.9⁢U/ml	NT-pro-BNP	0.03
		0.297	
RLN2 (pre-op)	24.5±5.5⁢pg/ml	RLN1	0.0003
		0.453	
CRP (post-op 24 h)	19.9±14.0⁢mg/dl	RLN1	0.03
		0.448	
CRP (post-op 48 h)	108.7±62.8⁢mg/dl	Cholinesterase	0.04
		0.401	
CRP (post-op 72 h)	120.3±53.4⁢mg/dl	Cholinesterase	0.02
		0.453	
PCT (pre-op)	0.13±0.1⁢ng/ml	RLN2	0.01
		0.487	
PVR (pre-op)	2.9±1.4⁢WU	RLN1	0.001
		0.555	
CI (post-op 24 h)	$3.2\pm 0.7L/\min/m{}^{2}$	RLN2	0.003
		0.919	
SVRI (post-op 24 h)	1441.3±392.3	RLN2	0.019
	Dynes/sec/cm^–5^/m^2^	−0.835	

*Invasive PAC and hemodynamic measurements were carried out upon referral for heart transplantation (pre-op) and 24 h after heart transplantation surgery (post-op). Bloods were drawn immediately before surgery and 24, 48, 72 h afterward (pre-op, pre-operatively; post-op, post-operatively; NT-pro-BNP, N-terminal pro b-type natriuretic peptide; RLN1, relaxin-1; RLN2, relaxin-2; CRP, C reactive protein; PCT, procalcitonine, PVR, pulmonary vascular resistance; CI, cardiac index; SVRI, indexed systemic vascular resistance).*

### Echocardiography and Invasive Hemodynamic Measurements

The results of transthoracic echocardiography can be seen in Table 1. All healthy subjects had normal LVEF, without any wall motion abnormalities or valve disease. Echocardiography parameters in the patient population were in keeping with HFrEF severely decreased systolic LV function (average LVEF 23.4 ± 5.5%) was observed. Left ventricular dimensions, as well as systolic and diastolic volumes were increased, showing dilation and remodeling of the left ventricle. Average right ventricular diameters were also dilated and TAPSE showed decreased longitudinal contraction of right ventricle. Our data show that CO were decreased and CI severely compromised in end-stage HF population. Interestingly, average values of pulmonary arterial pressures and pulmonary vascular resistance were increased, referring to chronic pathological remodeling in the pulmonary arterial vasculature, which is common in HFrEF. According the definition of pre-, post-capillary and combined pulmonary arterial hypertension most of our patients had combined pulmonary hypertension (pulmonary capillary wedge pressure: 21.76 ± 7.15 mmHg, Wood units: 2.69 ± 1.8, diastolic pulmonary arterial pressure: 20.65 ± 6.5 mmHg) in keeping with the long-standing LV dysfunction, maximal compensatory mechanisms and end-stage HF (Charalampopoulos et al., 2018).

Table 4 shows correlation of echocardiography and hemodynamic parameters with circulating RLN1 levels. RLN1 levels showed significant positive correlation with left ventricle inflow (E/A) parameters. Furthermore, circulating RLN1 levels showed significant negative correlation with a morphology parameter (left ventricular end-systolic diameter) and diastolic pulmonary artery pressure.

**TABLE 4:** Association of circulating RLN1 and morphology, function, and hemodynamic parameters of end-stage hearts with ischemic etiology and HFrEF.

**Parameter**	**Correlation coefficient**	***p*-value**
LVEF (%)	−0.023	0.879
E (cm/s)	0.243	0.147
E/A	**0.456**	**0.003**
TAPSE (mm)	−0.271	0.121
DCT (ms)	−0.094	0.608
LVEDD (mm)	−0.278	0.078
LVESD (mm)	−**0.373**	**0.023**
LVESV (ml)	−0.047	0.853
LVEDV (ml)	−0.035	0.883
RVEDD (mm)	0.119	0.489
horizontal RAD (mm)	0.283	0.094
vertical RAD (mm)	0.255	0.112
septum DD (mm)	0.010	0.956
PVR (Wood units = mmH⋅gmin/l)	−0.107	0.603
PCWP (mmHg)	−0.127	0.512
systolic PAP (mmHg)	−0.118	0.474
diastolic PAP (mmHg)	−**0.894**	**<0.001**
mean PAP (mmHg)	−0.143	0.392
CO (L/min)	0.036	0.817
CI (L/min/m^2^)	0.211	0.174

*Pearson’s correlation test was used. Echocardiography, PAC, and invasive hemodynamic measurements were obtained upon referral for heart transplantation list (CI, cardiac index; CO, cardiac output; DCT, deceleration time; DD, diastolic diameter; E, E wave; E/A, E wave/A wave; LVEDD, left ventricular end-diastolic diameter; LVEDV, left ventricular end-diastolic volume; LVEF, Left ventricular ejection fraction; LVESD, left ventricular end-systolic diameter; LVESV, left ventricular end-systolic volume; PAP, pulmonary artery pressure; PCWP, pulmonary capillary wedge pressure; PVR, pulmonary vascular resistance; RAD, right atrial diameter; RVEDD, right ventricular end-diastolic diameter; TAPSE, tricuspid annular plane systolic excursion). Bold values indicate the significant level at: *p* ¡ 0.05.*

### Increased RLN1 Levels Are Accompanied by Decreased Myocardial Fibrosis

**FIGURE 3:**
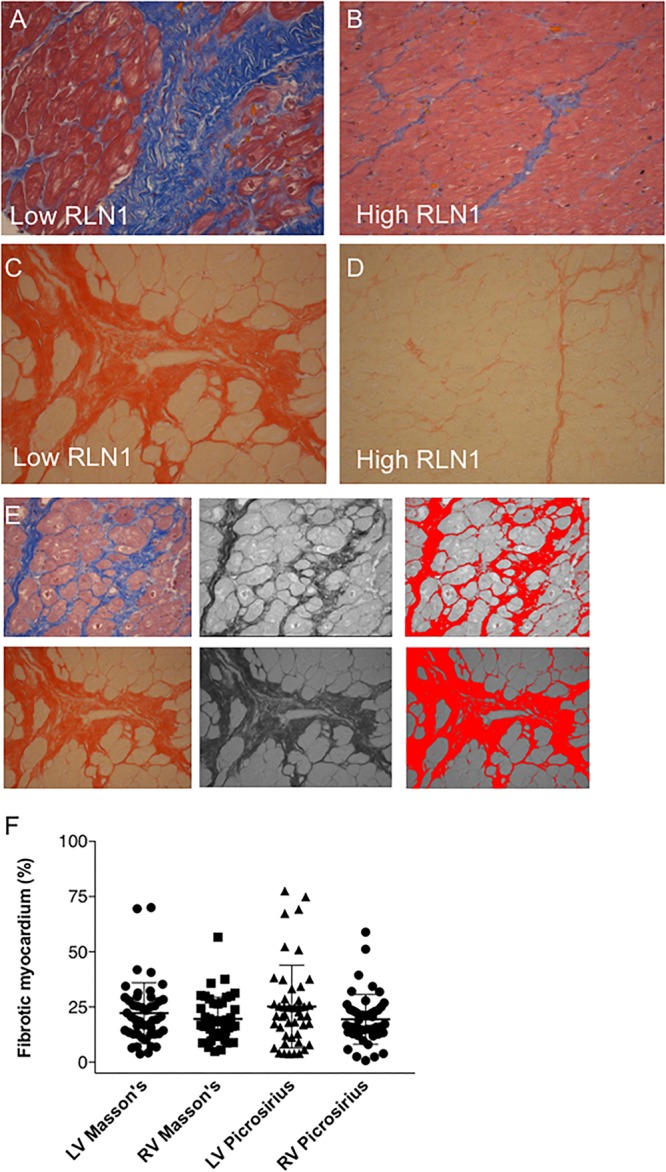
Quantification of myocardial fibrosis. Representative histology of **(A,B)** Masson’s trichrome and (**C,D**) picrosirius red staining. (**A,C**) Sections depict severe fibrosis of the left anterior segment of the left ventricle and (**B,D**) sections show mild myocardial fibrosis in the same area. Severely fibrotic tissues were derived from a patient with low Relaxin-1 level, while mild fibrosis was associated with high Relaxin-1. All myocardial tissues were collected upon explant of the failing heart in the operation theater and were immediately processed for histology. **(E)** Quantification of fibrotic tissue in histology sections was performed in ImageJ. Image processing comprised of color transformation followed by threshold adjustment and pixel assessments. **(F)** Whisker dot diagram show amount of myocardial fibrosis in percentage of the whole left ventricle/right ventricle, Masson’s trichrome and Picrosirius red staining and analyses (RLN1, Relaxin-1; LV, left ventricle; RV, right ventricle).

**FIGURE 4:**
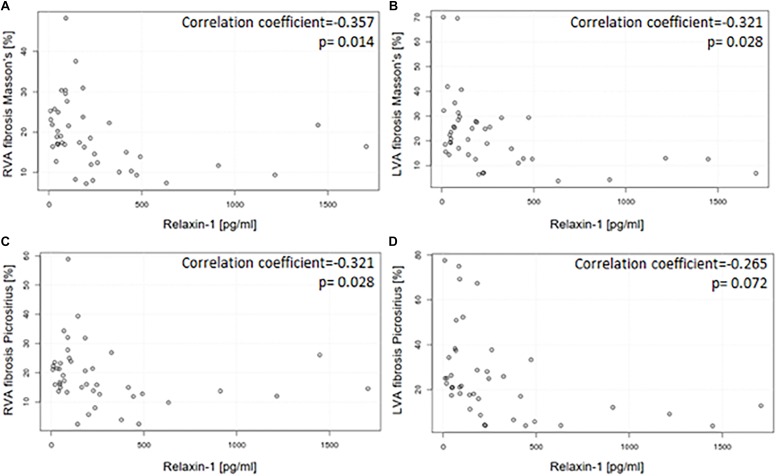
Correlation between circulating RLN1 levels and the extent of myocardial fibrosis in explanted hearts with ischemic etiology and HFrEF. Circulating RLN1 levels are associated with the extent of myocardial fibrosis both in the right **(A,C)** and left **(B,D)** ventricles. Quantification of myocardial fibrosis was assessed by Picrosirius red and Mason’s trichrome staining. Myocardial tissue was collected right after explant of the heart. RLN1 levels were measured from serum which was collected immediately before heart transplantation surgery. As statistic probe Pearson’s correlation test was used (LVA, anterior wall of left ventricle; RVA, anterior wall of right ventricle).

Figure 3 shows representative images of the patients with the high and low RLN1 level (Figure 3E). Extent of myocardial fibrosis was high in both ventricles in all patients with HFrEF (Figure 3F). There is statistically significant, inverse correlation between patients’ RLN1 levels and the extent of the fibrosis of both ventricles, suggesting that higher RLN1 levels are accompanied by smaller fibrotic areas (Figure 4).

### Gene Expressions

**FIGURE 5:**
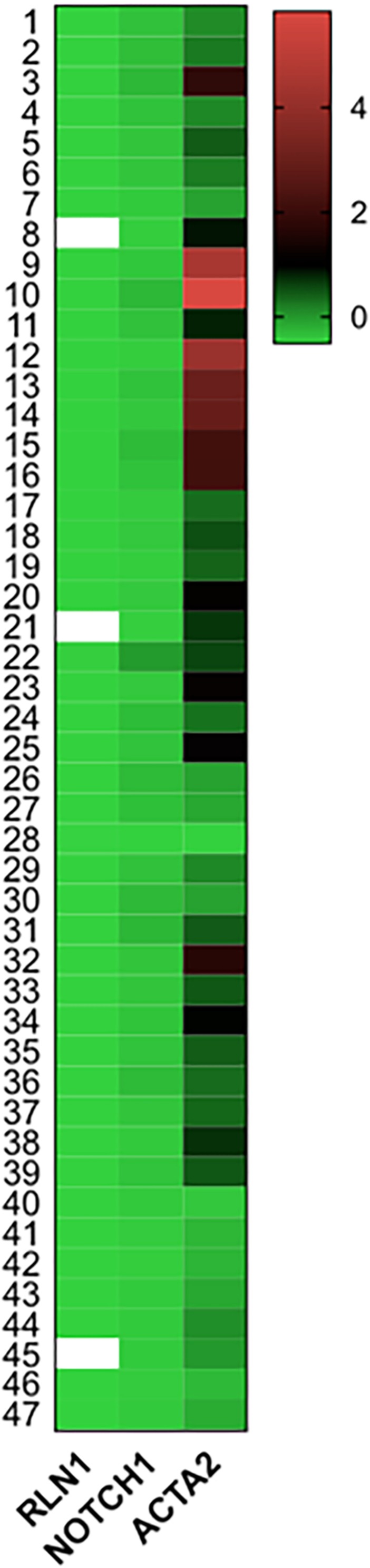
mRNA expressions of Relaxin-1, Notch-1, and ACTA2 in end-stage HFrEF and ischemic etiology. Gene expressions were measured from homogenized human myocardial septal samples. Tissue were collected and fresh frozen in operation theater right after the explant of the failing heart in heart transplantation surgery. Heat map shows expression of Relaxin-1 (RLN1), Notch-1, and ACTA2 (smooth muscle actin) mRNA levels normalized to GAPDH in z-score fashion. *n* = 47.

Figure 5 shows mRNA expressions of RLN1, Notch1 and ACTA2 (α-smooth muscle actin) in HFrEF patient population. Of note, RLN1 gene expression showed abundant expression, suggestive of with high and low expression profile as well. RLN1 and Notch1 mRNA expressions are suggestive of distinct signaling pathways which influence translation of relaxin proteins. ACTA2 levels were widely increased in our patient population referring to its strong regulatory role in the pathophysiology of HFrEF and myocardial remodeling. ACTA2 levels showed significant correlation with systemic vascular resistance post-operative at 48 h (*r* = 0.64, *p* = 0.03).

## Discussion

HF affects around 26 million people worldwide (Ponikowski et al., 2014). HF is a disease characterized by quick progression and high mortality (Schocken et al., 1992; Ho et al., 1993; Rodeheffer et al., 1993; Vasan et al., 1999; Cowie et al., 2000; Mosterd et al., 2001). The two main causes of death are sudden cardiac death and progressive pump failure (CONSENSUS Trial Study Group, 1987; SOLVD Investigators et al., 1991). The proper pathogenesis of ischemic myocardial remodeling is still obscure. In the literature, there is only a limited amount of data regarding the diastolic function of the left ventricle and the systolic function of the right ventricle in ischemic cardiomyopathy (Schinkel et al., 2004; Velazquez et al., 2011). The latter can play a prominent role in the prognosis of patients after heart-transplantation by influencing pulmonary circulatory conditions. Furthermore, the function of the right ventricle is important in the acute decompensation of the patients with chronic HF (Frea et al., 2016).

### Endogenous RLN Signaling Is Independent of Notch in Humans

Prior studies have shown that RLN increases coronary blood flow and counteracts in the pathophysiological changes of ischemic heart disease (Bani-Sacchi et al., 1995; Samuel et al., 2003). Exogenous administration of RLN proved to be effective in some non-cardiac conditions, such as in pulmonary and peri-bronchiolar fibrosis caused by bleomycin treatment, where collagen degradation was reported as a result of RLN administration (Pini et al., 2010, 2012; Royce et al., 2012). Anti-fibrotic properties of RLN have also been shown in skeletal muscle injury (Negishi et al., 2005; Mu et al., 2010). RLN can exert beneficial effect not only by influencing basal collagen expression but also by stimulating expression of extracellular matrix-degrading enzymes (Samuel et al., 2004a), as well as by inhibiting TGF-β1-induced fibroblast-myofibroblast transition (Mookerjee et al., 2009). In 2013 Sassoli et al. (2013) showed that RLN exerts its anti-fibrotic effect through the Notch-1-mediated inhibition of TGF-β/Smad3. Inhibition of the Notch-1 pathway promotes fibroblast-myofibroblast conversion (Bray, 2006; Fan et al., 2011). Sassoli et al. (2013) aimed to investigate the exact role of Notch-1 pathway in neonatal cardiac stromal cells in mice to show whether RLN has its anti-fibrotic effect through this signaling pathway. They proved that RLN exerted this effect through Notch-1 signaling. RLN was able to prevent the reduction of Notch-1 via TGF-β1 induction. After the administration of RLN together with DAPT (*N*-[(3,5-Difluorophenyl) acetyl]-L-alanyl-2-phenyl-glycine-1,1-dimethylethyl ester), that is an endogenous inhibitor of the Notch-1 pathway, RLN could not exert its effect, resulting in increased cardiac fibrosis. They have also found evidence that RLN (and Notch-1 pathway) influences fibrosis by interacting with the TGF-β-mediated signaling (Sassoli et al., 2013). However, these studies mainly focused on the action of exogenous RLN, but little was given to the investigation of endogenous RLN. Our studies were performed on human myocardial samples and interestingly we could not prove prominent signaling role of Notch-1 in RLN related effects, suggesting distinct signaling mechanisms in murine and human species. Given that TGF-β has a prominent role in cardiac remodeling, its activation undergoes in Notch-1 independent fashion in this HFrEF patient population (Barter et al., 2010). These can include matrix metalloproteinases, collagen-1, and collagen-3 related signaling (Chillakuri et al., 2012). Furthermore, relaxins affect renal fibrosis and its correlation with members of the renin-angiotensin-aldosterone system may also influence reverse remodeling. Our results proved high expression of ACTA mRNA suggestive of high volume of myocyte-fibroblast trans-differentiation. Of note, our studies investigated endogenous circulating and not exogenously administered relaxin in a human end-stage HF patient population. Given that Notch1 has homogenous expression within our patient population, presumably RLN acts not via the Notch-1 but rather distinct signaling pathways.

### Myocardial Remodeling and Hemodynamic Considerations of RLN Effects

In contrast with the RLN1 results, we found higher RLN2 levels in the control group, suggestive of their distinct role or specificity in the pathophysiology of HF. RLN2 supposed to have a pleiotropic effect in HF with preserved ejection fraction (HFpEF) acting via RXFP1 and glucocorticoid receptors (Dschietzig, 2019). Relaxins are related to the insulin hormone superfamily and proved to have different functional activities on the cardiovascular system. Importantly, relaxins have a strong vasoactive effect, characterized as vasodilation in isolated vessel experiments (Lian et al., 2018). The most potent vasodilatory factor amongst them is RLN2. Here in our experiments, the studied patient population (end stage HFrEF) is in a strong compensatory circulatory phase and centralization of blood flow. In this patient population, we have observed lower levels of RLN2, than in control subjects. We suppose this result is in direct relation with the pathophysiology changes of the circulation in end-stage HF. In keeping with the pathophysiology, end stage HF patients usually present with cold extremities, vasoconstriction on peripheries, and maximal centralization of the circulation. Healthy subjects maintain a steady state equilibrium with relatively high RLN2 levels, which underpin our findings. Recently, the RELAX-AHF trial enrolled 1161 patients with acute decompensated HF who received serelaxin (recombinant human relaxin-2) in acute HF (Teerlink et al., 2013). Serelaxin reduced average length of hospital stay and after 6 month of follow-up it significantly lowered the rate of cardiovascular death (HR 0.63, 95% CI 0.41–0.96) and all-cause mortality (HR 0.83, 95% CI 0.43–0.93). The RELAX-AHF-2 trial enrolled 6545 subjects with acute decompensated HF (Teerlink et al., 2017). In this study, there was no significant difference between the serelaxin and placebo groups neither in the lengths of hospital stay, nor in cardiovascular and all-cause mortality rates.

Hepatic failure is often caused by congestion accompanied by right heart dysfunction in HF patients. It is well-known that there is a negative correlation between serum cholinesterase level and liver function, decreased level means more severe hepatic condition. In keeping with this phenomenon, our data proved decreased cholinesterase levels in the patients with HFrEF when compared to healthy volunteers, suggesting poor liver condition and comprised right ventricular function in the HFrEF population. Another important hemodynamic observation is that post-operative vasoplegia is often seen and complicates the peri-transplantation period (Nemeth et al., 2018). In most of the cases the primary reason for vasoplegia remains unclear and thus the therapy is empiric and mostly symptomatic with high doses of vasopressors. In our study, elevated RLN1 levels were associated with significantly lower amount of myocardial adverse remodeling, fibrosis. Furthermore, higher RLN1 levels were linked with significantly higher E/A ratio, lower LVESD, and decreased diastolic PAP. Moreover, in our experiment, higher endogenous RLN1 levels were accompanied by significantly lower amount of myocardial fibrosis in patients with HFrEF. These results suggest that beyond a microscopic anti-fibrotic effect, RLN1 may also influence macroscopic LV remodeling. However, in this patient population RLN1 levels were not correlated with diastolic LV volumes, which can be in keeping with the end-stage dilation and volume overload of LVs in these patients. RLN1 may influence LVEDD in an earlier stage of HF, when LV reverse remodeling is still in a reversible phase. Interestingly, RLN1 affected the diastolic but not the systolic PAP. Clinical significance of these results lies in that RLN1 affects pulmonary circulation as well, in keeping with its anti-fibrotic effect in the lungs. Our study was limited to histology samples from human hearts and not lungs, but further investigations may reveal that RLN1 could be a therapeutic target in defined forms of pulmonary hypertension. Thorough clinical study should answer how diastolic and systolic PAP changes, mirroring RLN1 levels and the degree of pulmonary fibrosis.

Our findings are suggestive that circulating RLN1 levels significantly interfere with low cardiac output syndrome and post-operative hemodynamic changes in HFrEF, emphasizing a regulatory role in vasomotor functions. Given that RLN showed correlation with vascular hemodynamic measurements, liver and acute phase proteins (such as CRP and cholinesterase) we suggest that RLN may have and important regulatory role in hemodynamics mainly via acute phase proteins and other paracrine agents. We aim to validate these in a future clinical study in heart transplantation and/or MCS surgeries complicated by post-operative vasoplegia syndrome.

The role of RLN1 in the prognosis and therapy of HFrEF is widely investigated, but still a limited amount of data exists and the majority of these studies concentrate on the effects of exogenous RLN, while little is given to the investigation of endogenous RLN. Here, we investigated unique features of relaxin from human serum and explanted heart samples of patients with end-stage ischemic HF and this study permits direct and more accurate examination of myocardial fibrosis and effects of circulating RLN. Although current evidence-based use of renin-angiotensin system inhibitors, beta receptor blockers, statins, and aldosterone antagonists can delay cardiac remodeling, their effect is still unsatisfactory in terms of significant reversal of adverse remodeling (Krum et al., 2013). Therefore, we need to find and regulate (inhibit or activate) the factors responsible for the fibroblast-myofibroblast conversion in order to treat and prevent cardiac fibrosis more effectively. Our results promote the therapeutic use of RLN1 as an anti-fibrotic drug, but further studies are necessary to fully understand its complex intracellular signal pathways.

## Data Availability

The raw data supporting the conclusions of this manuscript will be made available by the authors, without undue reservation, to any qualified researcher.

## Ethics Statement

This study was carried out in accordance with the recommendation of the Hungarian Medical Research Council [ETT TUKEB 7891/2012/EKU (119/PI/12.) and TUKEB 73/2005] and their ethical approval of standard operation protocols of the Transplantation Biobank of the Heart and Vascular Center at Semmelweis University. The healthy subjects were assessed via the Budakalasz Health Examination Survey (ETT TUKEB 8424-0/2011-EKU). All subjects gave written informed consent in keeping with the Declaration of Helsinki.

## Author Contributions

JS and EG performed the data collection, molecular biology probes, wrote and edited the manuscript, and prepared the figures. EN performed the clinical data collection, edited the manuscript, and contributed to the biobanking. TR managed the biobanking, consulted the results, and edited the manuscript. GF and BM consulted the results and edited the manuscript. AN, MH-T, and JS contributed to the molecular biology probes. LK and ZB performed the data collection and managed the epidemiology study. All authors contributed to manuscript revision, read, and approved the submitted version.

## Conflict of Interest Statement

The authors declare that the research was conducted in the absence of any commercial or financial relationships that could be construed as a potential conflict of interest.

## Abbreviations

RLN: Relaxin
RLN1: Relaxin-1
RLN2: Relaxin-2
